# GMEmbeddings: An R Package to Apply Embedding Techniques to Microbiome Data

**DOI:** 10.3389/fbinf.2022.828703

**Published:** 2022-04-26

**Authors:** Christine Tataru, Austin Eaton, Maude M. David

**Affiliations:** ^1^ Department of Microbiology, College of Science, Oregon State University, Corvallis, OR, United States; ^2^ Department of Pharmaceutical Sciences, College of Pharmacy, Oregon State University, Corvallis, OR, United States

**Keywords:** microbiome, embedding, deep learning, machine learning, 16s sequencing

## Abstract

Large-scale microbiome studies investigating disease-inducing microbial roles base their findings on differences between microbial count data in contrasting environments (e.g., stool samples between cases and controls). These microbiome survey studies are often impeded by small sample sizes and database bias. Combining data from multiple survey studies often results in obvious batch effects, even when DNA preparation and sequencing methods are identical. Relatedly, predictive models trained on one microbial DNA dataset often do not generalize to outside datasets. In this study, we address these limitations by applying word embedding algorithms (GloVe) and PCA transformation to ASV data from the American Gut Project and generating translation matrices that can be applied to any 16S rRNA V4 region gut microbiome sequencing study. Because these approaches contextualize microbial occurrences in a larger dataset while reducing dimensionality of the feature space, they can improve generalization of predictive models that predict host phenotype from stool associated gut microbiota. The GMEmbeddings R package contains GloVe and PCA embedding transformation matrices at 50, 100 and 250 dimensions, each learned using ∼15,000 samples from the American Gut Project. It currently supports the alignment, matching, and matrix multiplication to allow users to transform their V4 16S rRNA data into these embedding spaces. We show how to correlate the properties in the new embedding space to KEGG functional pathways for biological interpretation of results. Lastly, we provide benchmarking on six gut microbiome datasets describing three phenotypes to demonstrate the ability of embedding-based microbiome classifiers to generalize to independent datasets. Future iterations of GMEmbeddings will include embedding transformation matrices for other biological systems. Available at: https://github.com/MaudeDavidLab/GMEmbeddings.

## 1 Introduction

Gut microbiomes can impact the physiology of their human hosts by modifying the availability of molecules in the environment or through direct interactions with host cells Ruff et al. (2020). The most commonly used and cost-effective method to observe microbiomes is 16S rRNA amplicon sequencing, which allows researchers to observe which bacterial species are present in an environment, their relative quantities, and their relative evolutionary distances to one another Johnson et al. (2019).

While 16S rRNA amplicon sequencing has many strengths and provides insight into general microbiome compositions, analysis of 16S data is often impeded by small sample sizes paired with massive feature spaces. This can lead to underpowered studies and spurious associations being detected Schloss (2018), Ioannidis (2005), Fan et al. (2012). While meta-analyses of microbiome datasets generally support associations between microbiome community structure and disease, these interactions are often relatively weak and confounded by inter-study and individual microbiome variation Sharpton et al. (2021), Sze and Schloss (2016), Holman and Gzyl (2019), Duvallet et al. (2017), Wirbel et al. (2021). In addition, 16S analysis generally treats amplicon sequence variants (ASVs) or operational taxonomic units (OTUs), also generally called taxa, as independent features, despite the complex network of known relationships between bacterial species that influence their function Albright et al. (2021), Shoaie et al. (2013). By reducing dimensionality while simultaneously analyzing 16S sequences in the context of co-occurrence and co-abundance patterns across studies, we can increase the generalizability of classifiers and gain insight into microbiome community function.

Embedding has emerged as a method in natural language processing to both decrease the dimensionality of the feature space as well as consider co-occurrence relationships between entities across corpuses of documents. Embedding algorithms produce a numerical vector representation of every feature, then datasets can be projected into this newly defined numerical space. Vector representations can be learned in multiple ways–here we use both GloVe and Principal Component Analysis (PCA) algorithms on American Gut Project (AGP) data to produce two sets of embedding vectors. GloVe is an algorithm designed for natural language processing which learns numerical representation of features by projecting a co-occurrence matrix between features into a lower dimensional space. In the case of natural language, these numerical vector representations of words can then be used to cluster words by their shared meanings and relationships (e.g., king–male = queen) Pennington et al. (2014). PCA is a method used frequently in ecology which learns numerical representation of features such that samples fall along the axes of highest variation across the dataset Karl (1901). To some extent, this method takes into account co-abundances between taxa across samples to learn a representation.

We used both of these algorithms to create embedding transformation matrices. Numerical representations of 48,279 ASVs found in 15,709 samples were learned, and representations were created in 50, 100, and 250 dimensions.

We present GMEmbeddings, an open-source R package that transforms 16s microbiome data (ASV counts) into an embedding space that captures information about taxa co-occurrence or co-abundance patterns. GMEmbeddings currently contains embedding vectors to enable embedding of 16S V4 reads from the human gut microbiome. While the presented embedding matrices are not meant to be used to transform counts from other 16S regions or other biomes, future iterations will include other sets of embedding vectors. The package also enables the ability to interpret the learned numerical representations in the context of microbial metabolic pathways.

Previous iterations of this work used only forward reads from the American Gut Project that were ∼ 150 bp long, resulting in less specificity and less coverage during the transformation into embedding space Tataru and David (2020). This iteration contains full length V4 reads ( ∼ 250 bp) to improve performance, and is additionally more accessible through use of a complete R package.

## 2 Methods

### 2.1 Making Embedding Transformation Matrix

#### 2.1.1 Data Collection

Fastq files were downloaded from ftp://ftp.microbio.me/AmericanGut/20nov2020-demultiplexed-data/. Only sequences from stool samples were kept. Each folder represents a study, and studies with less than 50 samples were removed. Of the 72 folders originally associated with the AGP, 50 folders were kept. All gzipped FASTQ files were then collected from each folder, totalling 43,256 individual files sharing a combined size of 113 Gigabytes of space. The files were then filtered using Cutadapt in order to remove primers from the sequences Martin (2011). We removed the 515F-806R primer pairs: GTGYCAGCMGCCGCGGTAA (Fwd V4), GGACTACNVGGGTWTCTAAT (Rev V4), GTGCCAGCMGCCGCGGTAA (Fwd V4), GGACTACHVGGGTWTCTAAT (Rev V4) McDonald et al. (2018). In an effort to keep only the most accurate samples available, further filtration was performed to retain only files containing over 5,000 sequence reads.

#### 2.1.2 Process Into ASVs

Fastq files were then processed using the DADA2 pipeline Callahan et al. (2016). In short, forward and reverse reads were trimmed to 140 base pairs, and maxEE and truncQ were set to 2. Reads that matched the phiX contamination database were removed Mukherjee et al. (2015). The error rates were then learned from the data, and later the core sample inference algorithm was applied to the filtered and trimmed sequence data. We then merged the forward and reverse reads together to obtain the full denoised sequences and removed any chimeras from the data. Lastly, bloom sequences obtained from the following link were removed: https://github.com/knightlab-analyses/bloom-analyses/blob/master/data/newbloom.all.fna.

#### 2.1.3 Filter for Prevalence

After completing the quality filter and trimming steps in the DADA2 pipeline, we created a sequence table. The entries in the sequence table represented counts of the number of times the sequence read was detected in each of the samples. In total, there were unique 898,853 ASVs and 15,706 samples (merged forward and reverse reads). However, many of these ASVs had low rates of occurrence among the samples, so further filtering was done to remove reads that were detected in 10 or fewer samples. Filtering for blooms and prevalence reduced the size of our sequence table from 898,855 ASVs to 48,279 ASVs.

#### 2.1.4 Calculate Co-Occurrence

Next, we created an ASV co-occurrence text file. Each line in the file contained the full length ASV sequence of all ASVs in one sample. The final file contained 15,706 lines, with one for every sample. In essence, we could think of each sample as having a specific sentence/catchphrase, and each line in this file contained the catchphrase of one sample. However, instead of words, each catchphrase was composed of the genetic sequences observed in each sample. This is the format for input files to the GloVe software (version 0.2). Pennington et al. (2014).

#### 2.1.5 GloVe Algorithm

The GloVe algorithm was then applied to our co-occurrence file in order to create an embedding transformation matrix of a set size Pennington et al. (2014). GloVe stands for global vectors for word representation and is an unsupervised learning algorithm to generate word embeddings from aggregated global word-word co-occurrence data. When the algorithm is applied to ASV sequences instead of words, the result is a vector representation for each ASV in the set that represents co-occurrence patterns. Cosine distances between vectors represents probability of co-occurrence of the corresponding ASVs. Short distances between sequences represent higher probabilities of ASVs sharing co-occurrence patterns, while greater distances represent lower probabilities of sequences sharing co-occurrence patterns. Distances are normalized by how frequently ASVs occur overall. The algorithm was run three times with output vector sizes of 50, 100, and 250.

#### 2.1.6 PCA Algorithm

The PCA transformation matrices were obtained using SVD decomposition on the sample by ASV count table, and taking the (*V* ^*T*^) matrix. Prior to decomposition, ASV count vectors were mean centered and scaled to have a variance of 1, to avoid issues with heteroskedasticity. R/making_embedding_transformation_matrix/make_PCA_transformation_matrix.py.

#### 2.1.7 Creating BLAST Database for ASVs in Embedding Database

The ASV sequences from the GloVe output were then used to create a FASTA formatted file. We then used the makeblastdb functionality of BLAST (Basic Local Alignment Search Tool) to generate a database based on the nucleotide sequences in our FASTA file. The database is used to check nucleotide sequences from other studies against our “embedding database” sequences. BLAST database files can be found here: https://files.cgrb.oregonstate.edu/David_Lab/microbiome_embeddings/blastdb_fullseq/.

### 2.2 Transforming Query Data Into Embedding Space

**FIGURE 1:**
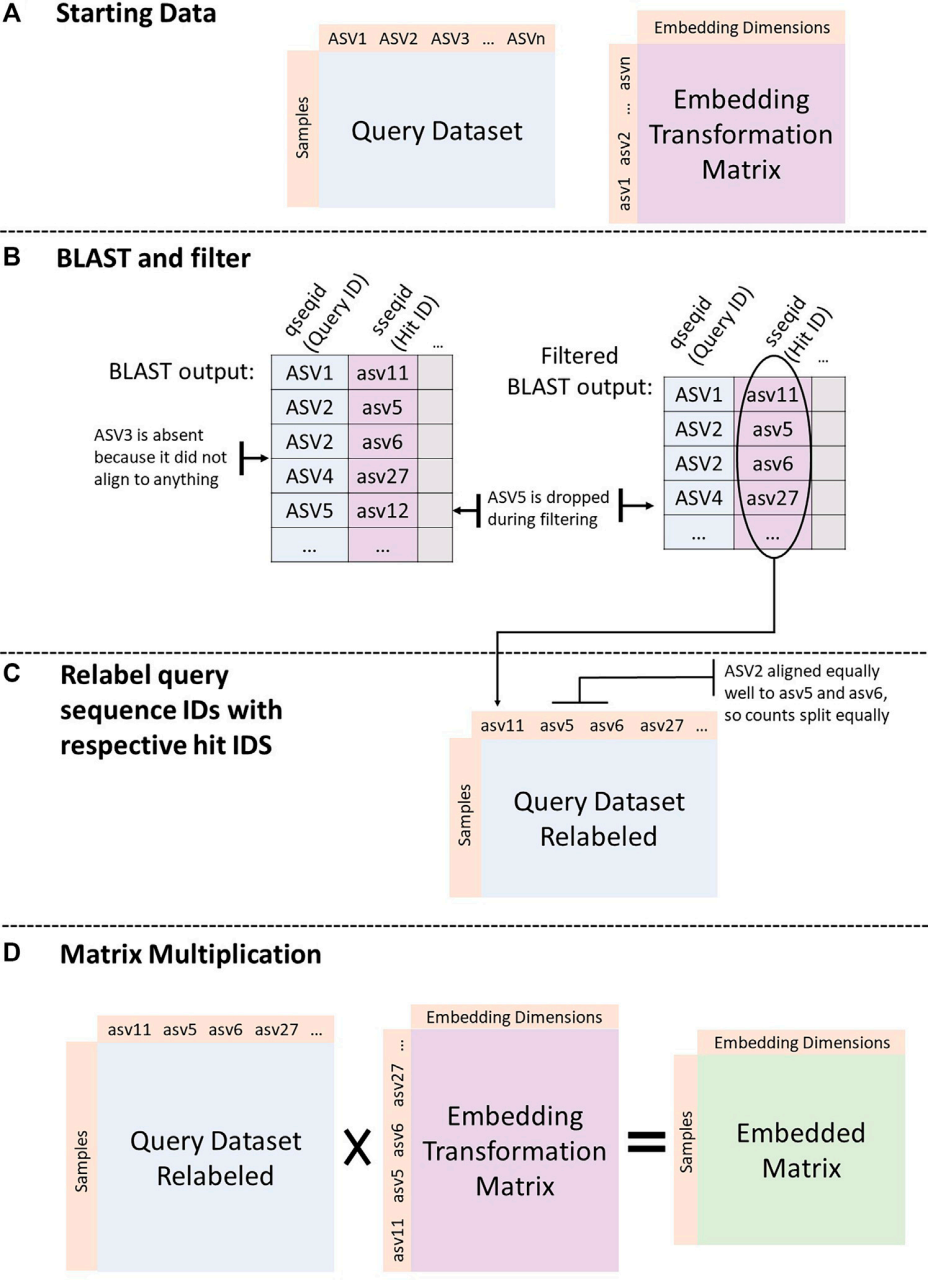
Embedding a dataset. **(A)** Start with query dataset (sample by ASV counts) and an embedding transformation matrix (either from GloVe or PCA run on the American Gut Project data). **(B)** BLAST ASV sequences from the query dataset against the sequences in the transformation matrix. Filter the BLAST output to include only top hits (min E-value, max percent identity, and max alignment length) per query sequence. **(C)** Assign ids from BLAST hit column to query sequences. If there are more than one best hit, split counts from original query sequence between all best hits equally. **(D)** Matrix multiply the query count table with the embedding transformation matrix.

Figure 1 shows the process implemented by the GMEmbeddings package to transform any query ASV count table into embedding space.

#### 2.2.1 BLAST Alignment

To embed a query sequence table, we first created a corresponding FASTA file for the ASVs in that study. We then used BLAST to obtain all hits for each query sequence against the sequences in the embedding database:


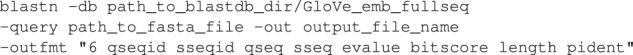


#### 2.2.2 Filter BLAST Output

We filtered the BLAST hits to include only the top match per query sequence (lowest E-value, highest percent identity, and highest length of alignment). We kept matches with a maximum E-value threshold of 1*10^−40^. Using a 97% similarity threshold, the maximum E-value and minimum length of alignment observed and accepted are available in Table 1. See R/making embedding transformation matrix/scripts/filter blast hits.sh.

**TABLE 1:** Maximum E-value and minimum length of alignment that were accepted in aligning sequences from each dataset to embedding transformation sequences.

Dataset	Max E-value accepted	Min. Length of alignment accepted
Halfvarson	3.72e-47	89
Schirmer	2.41e-86	133
M3	4.91e-125	129
Pilot	9.04e-122	110
Baxter	2.11e-133	110
Zeller	2.11e-133	121

#### 2.2.3 Relabel Query Sequence Ids With Respective Hit IDs

We relabeled query sequences with their respective hits in the embedding database. If the query sequence had only one top hit, we replaced its label with the label from the embedding database. If the sequence had multiple hits, we split its counts evenly among all of the top hits. If the sequence had over 100 hits that are all tied, it was dropped in an effort to increase the specificity of the method. If a query sequence had no hits, it was dropped. We also removed any sequences from the embedding transformation matrix that were never included as a top hit for any query sequence.

#### 2.2.4 Matrix Multiplication

After the above processing, the column space of the query count table matched the rowspace of the embedding transformation matrix. We then took the dot product between the two matrices to obtain the embedded form of the query count table. In the final embedded table, rows were samples and columns were dimensions in embedding space. Ultimately, the embedded form of a matrix represents the original samples transformed into a mathematical space, taking into account the co-occurrence patterns of ASVs across a population.

### 2.3 Machine Learning Process

We trained seven random forest models per dataset to predict phenotype, one using normalized read counts, three using GloVe embedded data at 50, 100, and 250 dimensions, and three using PCA embedded data at 50, 100, and 250 dimensions.

Model feature spaces had to match between training and testing sets, so some modification of feature spaces was required:

1) For the model on normalized read counts, we included only the ASVs that were present in both datasets. We performed a BLAST alignment between the query dataset and AGP sequences using a 100% sequence similarity cutoff, and assigned the ASV full length sequences from AGP to the secondary dataset (similar to the process of embedding without matrix multiplication). Only the best hits were considered from the resulting BLAST alignment after imposing the 100% similarity cutoff. Read counts from the secondary dataset were split equally among all the tied best hits in the AGP data.
2) For the models based on embedded data, we followed the procedure outlined above in “Transforming Query Data into Embedding Space”.

Prior to being fed to a machine learning model, all data was normalized using an inverse hyperbolic sin function, ( $\sin^{-1}\left(x\right)=\log\left(x+{\left(x^{2}+1\right)}^{1/2}\right)$ , which mimics the function log(2*x*) almost exactly, except for behavior near 0. Below 1, the log function returns a negative value, and is undefined at 0. In contrast, inverse hyperbolic sin does not fall below 0 when the argument is low, and is defined as 0 at 0 Burbidge et al. (1988), Sankaran and Holmes (2019). This function allows log transformation of counts without the addition of pseudocounts.

All models were trained entirely on one large dataset and tested on an independent dataset, paired as follows (AGP: Halfvarson, AGP: HMP2, M3: Pilot, Baxter: Zeller). Datasets are described below. We used random forest predictive models, and set maximum tree depth to the square root of the number of input features and the number of trees to 100. Classes were weighted inversely to their a priori probabilities in the training dataset $\left(pos\text{\_}weight=\frac{N}{N_{pos}}\right)$ . For example, if the positive class is represented by 5% of the training samples, the weight on the positive class for the training classifier is 20, and the weight on the negative class is 1.

### 2.4 Metabolic Pathway Correlation

In order to interpret the dimensions that define embedding spaces, we correlated each dimension in embedding space to all prokaryotic metabolic pathways available in the KEGG database Kanehisa et al. (2015). An infographic describing the process is available in Supplementary File S1. First, we created a binary pathway (ko id) by gene (KO id) table describing which genes are present in which metabolic pathways using the KEGGREST API in R (A). Then, we created a matrix of gene (KO id) by ASVs by using PICRUSt Langille et al. (2013) (B). We multiplied the pathway by gene table (A) with the gene by ASV table (B) to obtain an ASV by pathway table (C), where higher values suggest a higher presence of a pathway in that organism. We then calculated the Spearman correlation between all columns of these two matrices to obtain a pathway by dimension correlation matrix. These values can be used to interpret dimensions in a biological context.

### 2.5 Test Dataset Descriptions

#### 2.5.1 American Gut Project

In the American Gut Project (AGP) dataset, the majority of samples come from participants residing in the United States (*n* = 6,634) and the United Kingdom (*n* = 2,071), with a small number of samples generated from people living in other countries and territories. Participants in the United States inhabit largely urban areas (*n* = 7,317), with rural (*n* = 29) and mixed (*n* = 98) communities (2010 U.S. Census data based on participant ZIP codes) contributing in much smaller numbers. These participants also span a wide range of ages, race, and ethnicity. The read length of each sequence was around 150 base pairs which, when merged, resulted in a read length of 250 base pairs.

In the present study, we used a subset of 15,709 samples that were part of cohorts with > 50 samples in the consortium. These samples contained a collective 898,855 ASVs. We removed ASVs present in less than 10 samples, and 50,425 ASVs remained, each 253 base pairs in length.

#### 2.5.2 Halfvarson

The Halfvarson dataset Halfvarson et al. (2017) consists of 683 samples taken from 118 patients at multiple timepoints. Microbiome composition for each sample is ascertained by sequencing the V4 region of the 16S rRNA gene for a total of 248 million 16S rRNA gene amplicons and a total of 38,513 unique amplicon sequence variants (ASVs) at a read length of 253 bp.

In the present study, we filtered to include only samples with the diagnoses Crohn’s disease (CD), ulcerative colitis (UC), and healthy control (HC). We used 608 of these samples from 118 patients (220 CD, 290 UC, and 54 HC samples). When embedding using a 97% similarity threshold, 15,998 ASVs (61%) from the Halfvarson dataset aligned to some read in the embedding dataset (Supplementary File S2). Each query sequence was aligned to a mean of 10.4 and a median of 2 embedding sequences (Supplementary File S3). These same statistics applied to the PCA transformed data.

#### 2.5.3 HMP2

The HMP2 study Lloyd-Price et al. (2019) follows 132 subjscts for 1 year to generate longitudinal molecular profiles.

In the present study, we used only the 16S samples from the HMP2 study consisting of 197 samples taken from 83 individuals sampled at multiple timepoints (111 CD, 44 UC, 42 HC). This subsetted dataset contained a total of 5,869 unique ASVs at a length of 253 bp. When embedding using a 97% similarity threshold, 4,977 ASVs (85%) from the HMP2 dataset aligned to some read in the embedding dataset (Supplementary File S2). Each query sequence was aligned to a mean of 7.8 and a median of 2 embedding sequences (Supplementary File S3). These same statistics applied to the PCA transformed data.

#### 2.5.4 M3

The M3 dataset Tataru et al. (2021) consists of 432 total samples from 72 age-matched sibling pairs. The pairs included one sibling diagnosed with ASD and the other who is developing typically (TD). The participants were between the ages of 2 and 8 years old. Researchers recorded 331 diet and lifestyle variables for each individual participating in the study. For each sample collected there were an additional 100 variables detailing lifestyle and dietary variables recorded. Samples were collected across the United States. Before filtration, the average depth of reads per sample measured 157,103 nucleotides (with a minimum of 23,321 and maximum of 996,530). The dataset contains a total of 5,265 ASVs (16S V4) at a length of 233 bp.

In the present study, all samples from the M3 dataset were used. When embedding using a 97% similarity threshold, 4,555 ASVs (87%) from the M3 dataset aligned to some read in the embedding dataset (Supplementary File S2). Each query sequence was aligned to a mean of 2 and a median of 1 embedding sequences (Supplementary File S3).

#### 2.5.5 Pilot

The dataset obtained from the Pilot study David et al. (2021) contained 117 samples, of which, 60 were considered autism spectrum disorder (ASD) and 57 were controls. The population in the study consisted of age-matched sibling pairs between the ages of 2 and 7 years old, where the siblings needed to be within 2 years of each other. Of the 117 child subjects, there were 55 sibling pairs, two sibling pairs accompanied by a third sibling with autism, and 5 singleton samples. Samples were collected from 24 states: California, Colorado, Florida, Georgia, Hawaii, Illinois, Indiana, Massachusetts, Maryland, Michigan, Minnesota, Missouri, North Carolina, Nebraska, New Jersey, Nevada, New York, Ohio, Pennsylvania, Tennessee, Texas, Utah, Washington, and Wisconsin. The dataset contains a total of 1,664 ASVs (16S V4) at a length of 233 bp.

In the present study, all samples from the Pilot dataset were used. When embedding using a 97% similarity threshold, 1,500 ASVs (90%) from the pilot dataset aligned to some read in the embedding dataset (Supplementary File S2). Each query sequence was aligned to a mean of 1.8 and a median of 1 embedding sequences (Supplementary File S3).

#### 2.5.6 Zeller

The Zeller dataset Zeller et al. (2014) consists of three populations of participants: 129 colonoscopy patients from a French hospital (53 CRC, 42 adenoma, and 61 controls), 38 colorectal cancer patients from a German hospital, and 5 healthy individuals living in Germany. A subset of these participants were chosen for fecal sample 16s sequencing by the original authors for stool 16S sequencing.

The present study used 75 control and 41 CRC samples, and this set of samples contained a total of 6,968 unique ASVs at a length of 253 bp. When embedding using a 97% similarity threshold, 5,581 ASVs (80%) from the Zeller dataset aligned to some read in the embedding dataset (Supplementary File S2). Each query sequence was aligned to a mean of 1.2 and a median of 1 embedding sequences (Supplementary File S3).

#### 2.5.7 Baxter

The Baxter dataset Baxter et al. (2016) contains participants of ages 29–89 years with a median of 60 years. All patients were asymptomatic and were excluded if they had undergone surgery, radiation, or chemotherapy for current CRC prior to baseline samples or had inflammatory bowel disease, known hereditary non-polyposis CRC, or familial adenomatous polyposis. Colonoscopies were performed and fecal samples were collected from participants in four locations: Toronto (ON, Canada), Boston (MA, United States), Houston (TX, United States), and Ann Arbor (MI, United States).

The present study used 314 samples, (187 control and 127 CRC).

When embedding using a 97% similarity threshold, 7,879 ASVs (88%) from the Baxter dataset aligned to some read in the embedding dataset (Supplementary File S2). Each query sequence was aligned to a mean of 1.33 and a median of 1 embedding sequences (Supplementary File S3).

### 2.6 Metrics

#### 2.6.1 Precision

Precision is an indicator of a model’s performance and refers to the number of true positives divided by the total number of positive predictions. Total number of positive predictions can be found by summing the number of true positives with the number of false positives.

$precision=\frac{\left(truepositives\right)}{\left(truepositives\right)+\left(falsepositives\right)}$

#### 2.6.2 Recall

Recall gives indication of positive samples that the model has missed. It is calculated by dividing the number of true positives found by the model by the total number of positive samples that could have been made. The number of possible positive samples is the sum of true positives and false negatives.

$recall=\frac{\left(truepositives\right)}{\left(truepositives\right)+\left(falsenegatives\right)}$

#### 2.6.3 F1

The F1 score is the weighted average of precision and recall. It takes both false positives and false negatives into account and tells us a model’s performance on a dataset. A perfect model would have an F1 score of 1.

$F1Score=\frac{2*\left(recall\right)*\left(precision\right)}{\left(recall\right)+\left(precision\right)}$

## 3 Results

From the sequence counts from the American Gut Project (AGP), we created GloVe and PCA based embedding transformation matrices at 50, 100, and 250 dimensions. We then projected the sequence tables from six independent datasets, as well as that from the AGP, into both GloVe and PCA spaces. We then trained random forest predictive models to predict host phenotype using microbiome data in one of seven forms (GloVe embedded at 50, 100, and 250 dimensions, PCA embedded at 50, 100, and 250 dimensions, and normalized ASV counts). For each phenotype of inflammatory bowel disease (IBD), autism spectrum disorder (ASD), and colorectal cancer (CRC), models were trained on one dataset and tested on an independent set with no fine-tuning. No other metadata about samples was included in addition to microbiome data.

### 3.1 Inflammatory Bowel Disease Prediction

**FIGURE 2:**
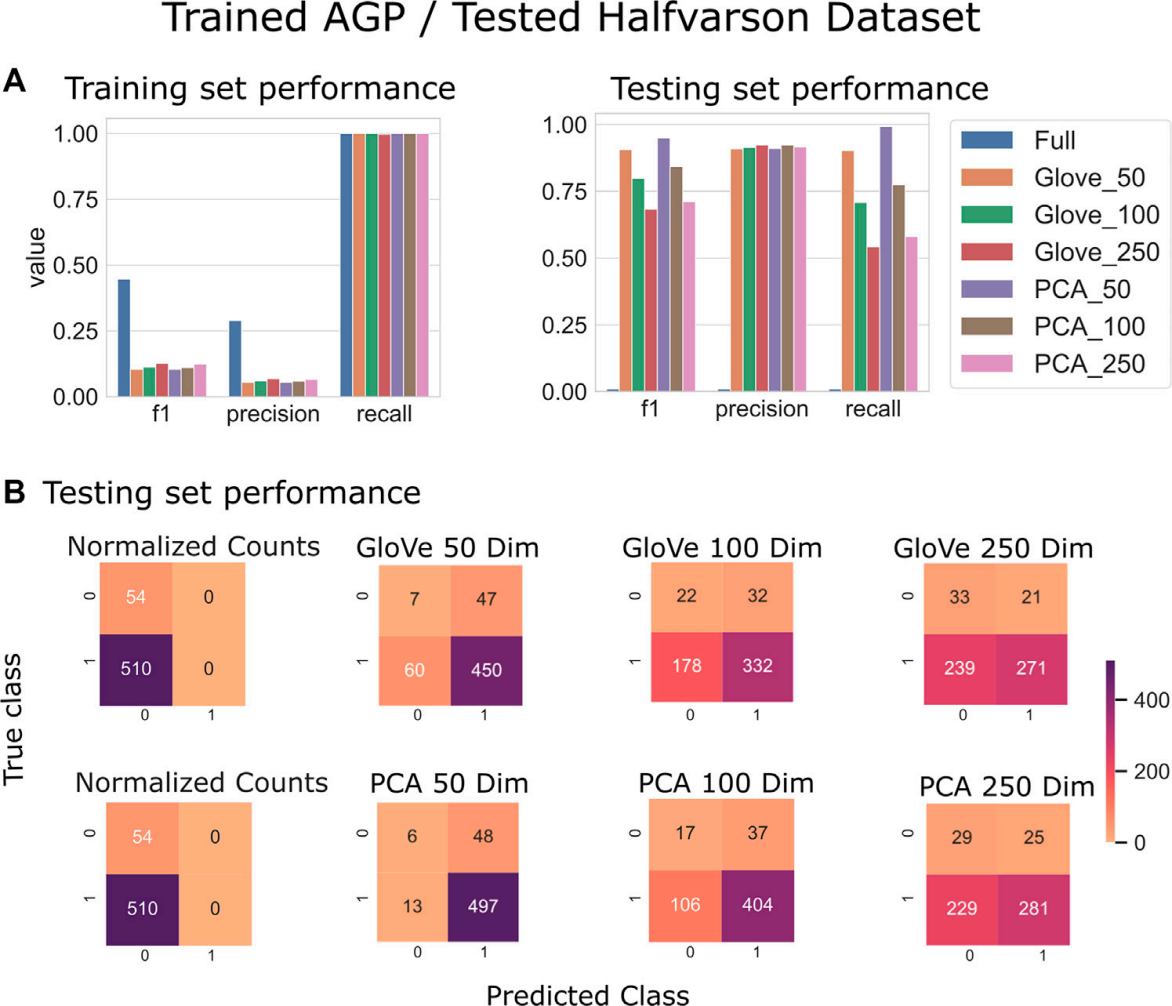
Predicting IBD: Model trained on AGP data and tested on Halfvarson data. **(A)**: Models built using GloVe embedded data, PCA embedded data (50, 100, or 250 dimensions), or normalized ASV counts performance on training and testing sets. **(B)**: Confusion matrices showing the distribution of correct to predicted classes on the testing dataset.

Random forest models were trained on the American Gut Project data, then tested on both the Halfvarson and HMP2 datasets Halfvarson et al. (2017), Lloyd-Price et al. (2019) to predict host phenotype of “healthy control” *vs*. “inflammatory bowel disease” which included Crohn’s disease and ulcerative colitis. On the Halfvarson test dataset, models that used normalized ASV counts (full) had a higher training performance but much lower testing performance than any of the other methods, implying an overfit model (Figure 2; Table 2). Similarly, while larger models using 250 dimensions generalized to a testing set less well (f1 = 0.68–0.71), small models using only 50 dimensions were able to generalize much more effectively (f1 = 0.9–0.95). GloVe and PCA embedding methods exhibited largely similar performance, regardless of the choice of sequence alignment threshold (Table 2, Supplementary File S4).

**TABLE 2:** Performance metrics of models trained on AGP data and tested on Halfvarson data using a 97, 99 and 100% sequence similarity threshold. 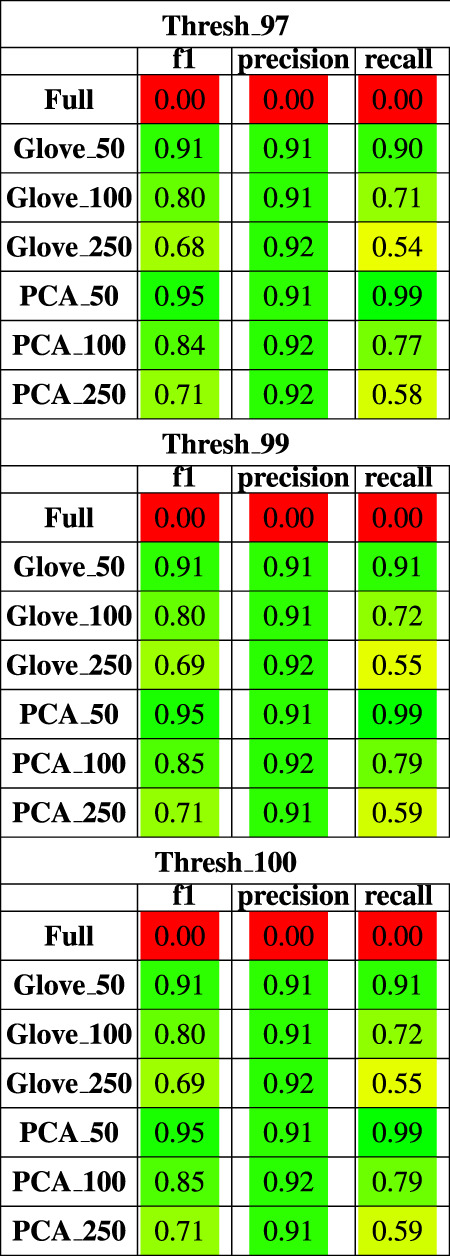

**FIGURE 3:**
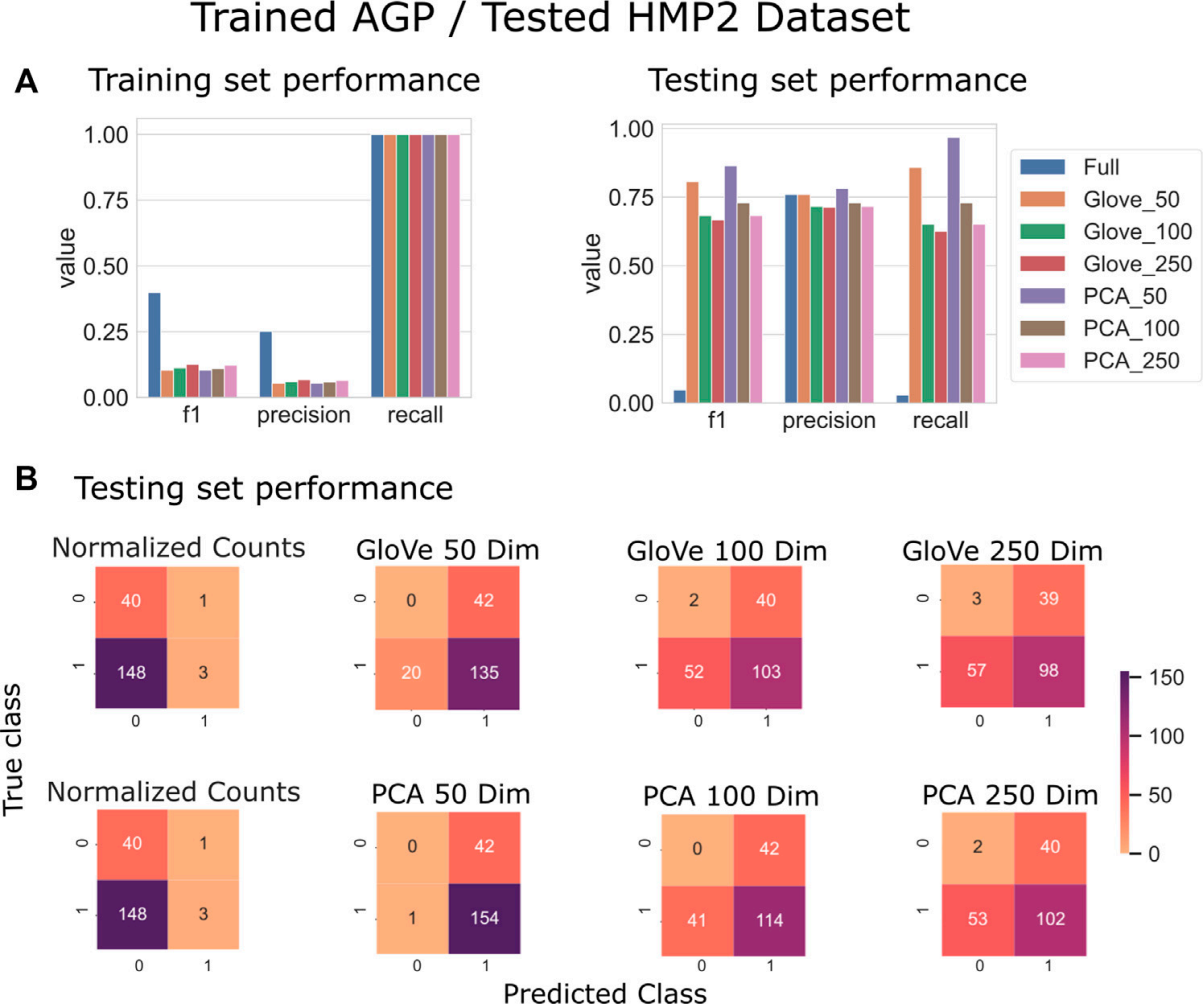
Predicting IBD: Model trained on AGP data and tested on HMP2 data. **(A)**: Models built using GloVe embedded data, PCA embedded data (50, 100, or 250 dimensions), or normalized ASV counts performance on training and testing sets. **(B)**: Confusion matrices showing the distribution of correct to predicted classes on the testing dataset. (full).

On the HMP2 test dataset, a similar phenomenon emerged. The full model trained well but failed to generalize well to the testing dataset, and the larger embedding-based models performed less well than smaller embedding-based models (Figure 3). Increasing sequence similarity threshold resulted in removing more original sequences (Supplementary File S3), and in this case, decreased overall performance considerably (Supplementary File S5, Table 3). There was similar performance between GloVe and PCA embedding-based models when using a 97% sequence similarity threshold, but PCA based methods maintained a higher performance as similarity threshold increased, as compared to GloVe based models (Supplementary File S5, Table 3).

**TABLE 3:** Performance metrics of models trained on AGP data and tested on HMP2 data using a 97, 99 and 100% sequence similarity threshold. 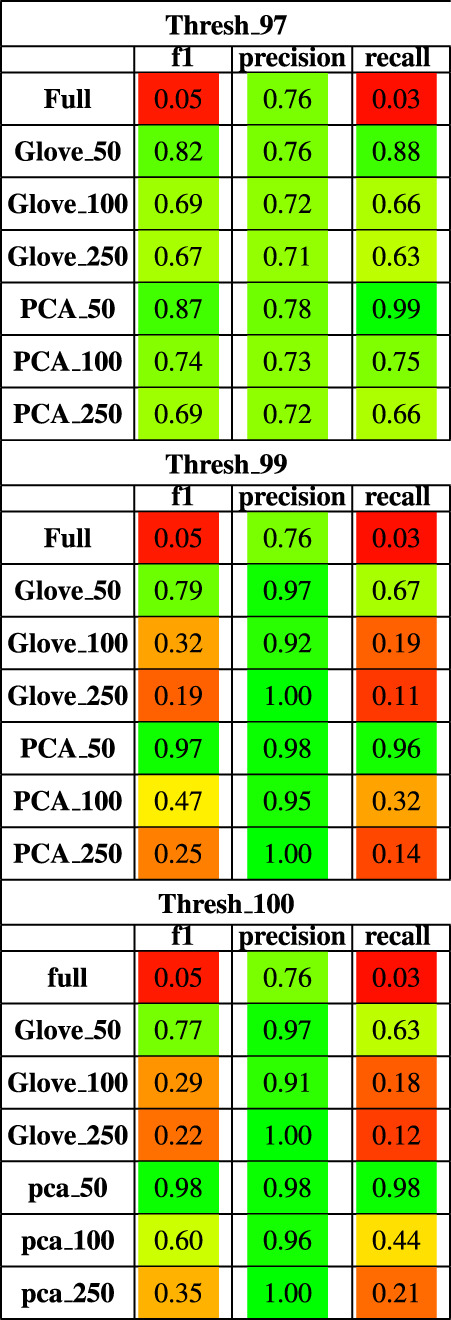

### 3.2 Austism Spectrum Disorder Prediction

**FIGURE 4:**
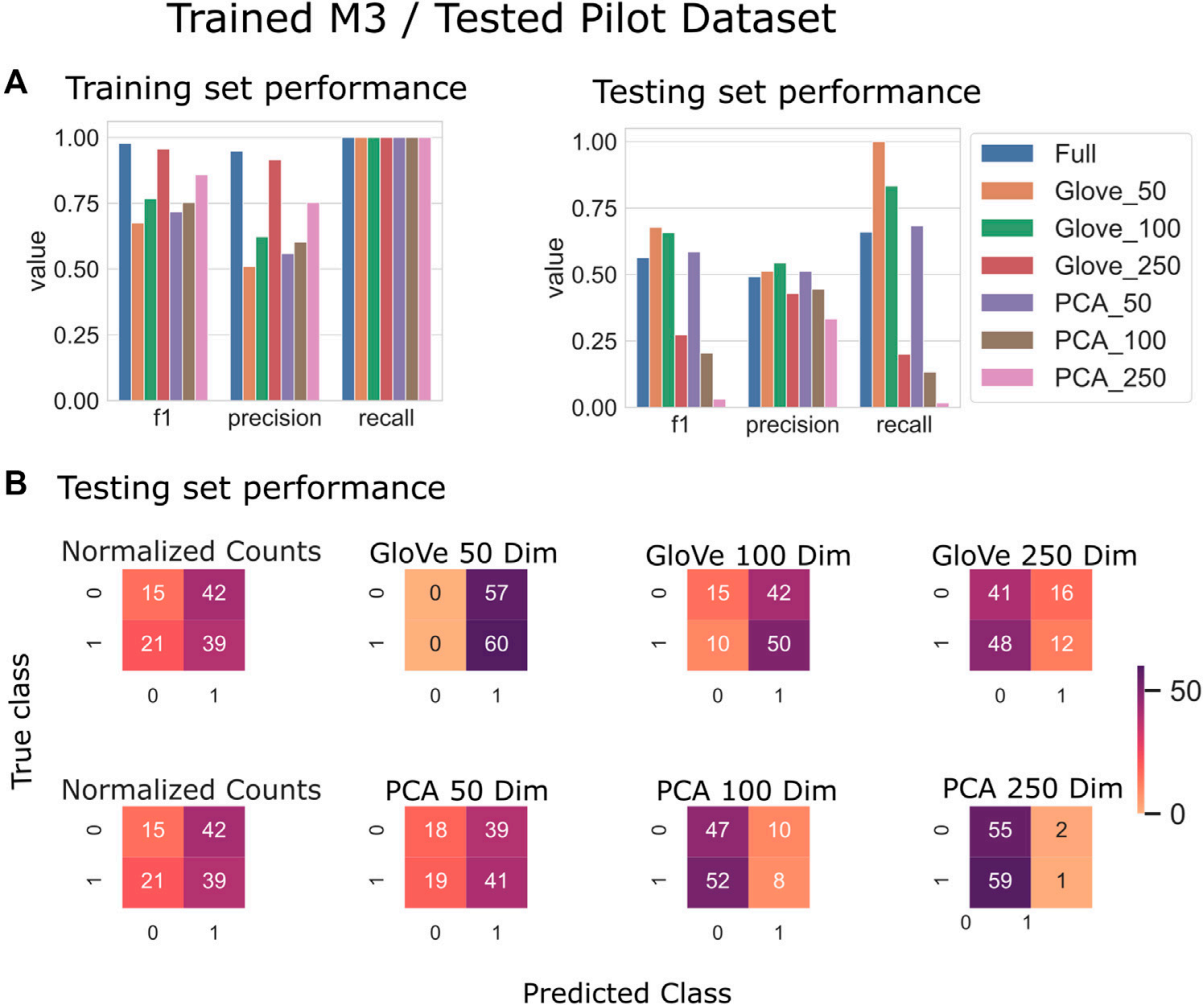
Predicting autism spectrum disorder: Model trained on the M3 dataset and tested on the Pilot dataset. **(A)**: Models built using GloVe embedded data, PCA embedded data (50, 100, or 250 dimensions), or normalized ASV counts performance on training and testing sets. **(B)**: Confusion matrices showing the distribution of correct to predicted classes on the testing dataset.

Random forest models were trained on the M3 dataset and tested on the Pilot dataset (see Test Dataset Descriptions) Tataru et al. (2021), David et al. (2021) to classify the phenotype of participants with autism spectrum disorder and their typically developing siblings. While the full model outperformed other models during training, it obtained an F1 score of 0.56 in testing, while the GloVe_50, GloVe_100 models obtained higher F1 scores of 0.67, 0.66 respectively (Figure 4; Table 4). Increasing sequence similarity threshold improved the performance of GloVe_250 and PCA_100 models, and did not significantly effect other models (Supplementary File S6).

**TABLE 4:** Performance metrics of models trained on M3 data and tested on Pilot data using a 97, 99 and 100% sequence similarity threshold. 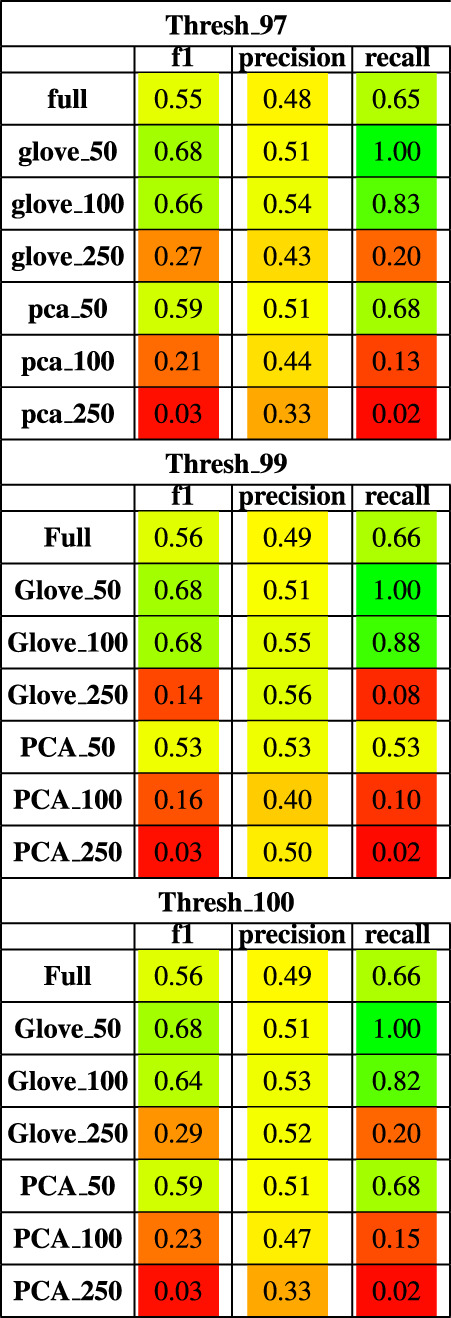

### 3.3 Colorectal Cancer Prediction

**FIGURE 5:**
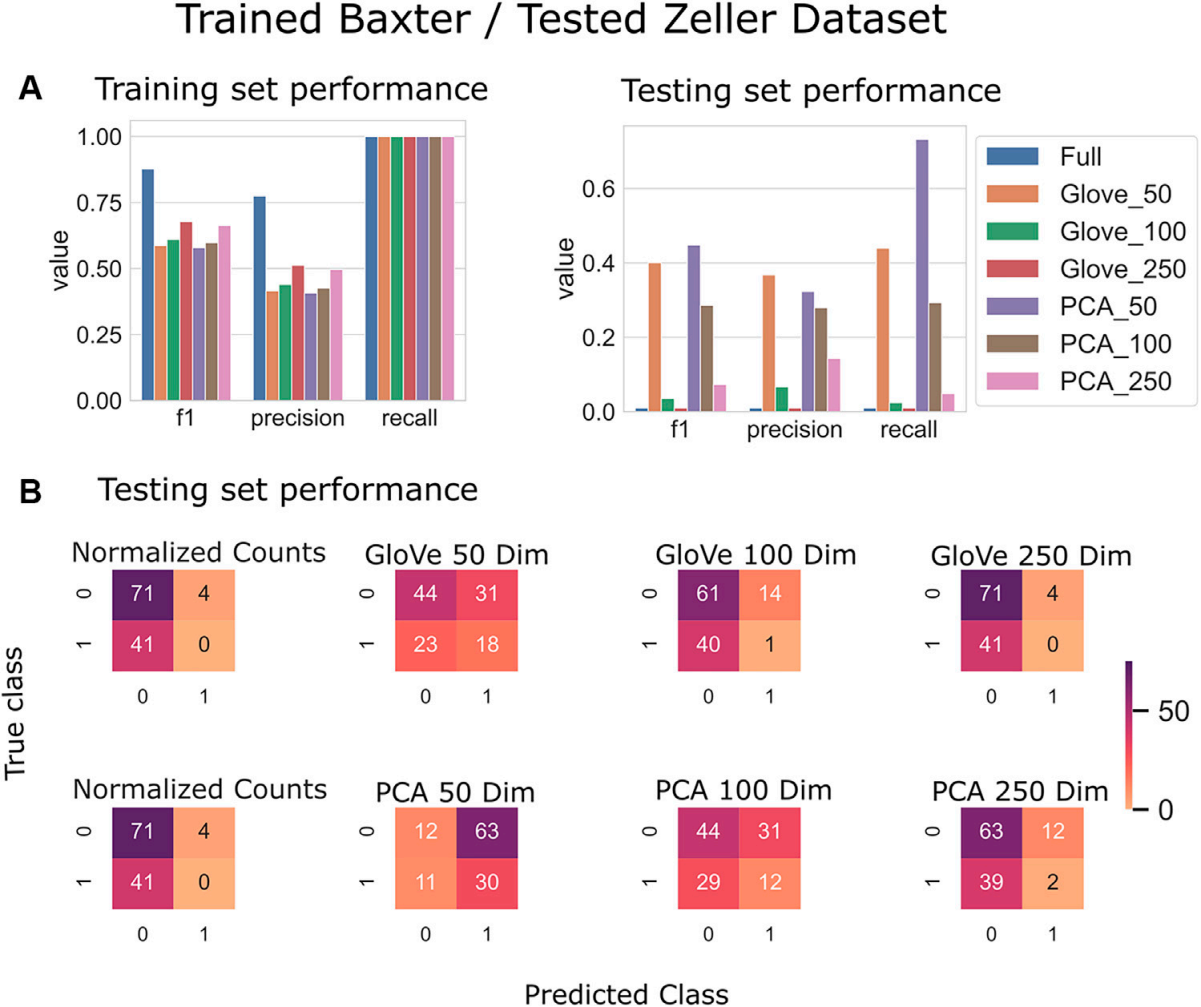
Predicting Colorectal Cancer: Models trained on the Baxter dataset and tested on the Zeller dataset. **(A)**: Models built using GloVe embedded data, PCA embedded data (50, 100, or 250 dimensions), or normalized ASV counts performance on training and testing sets. **(B)**: Confusion matrices showing the distribution of correct to predicted classes on the testing dataset.

Random forest models were trained on the Baxter dataset and tested on the Zeller dataset (see Test Dataset Descriptions) Baxter et al. (2016), Zeller et al. (2014) to classify the phenotype of participants with colorectal cancer *vs*. healthy controls. The full model had higher training performance but failed to generalize to the test set, and this trend repeated in the models built on more features in both GloVe and PCA based models. The highest performing models were PCA_50 and GloVe_50 with F1 scores of 0.45 and 0.4 respectively (Figure 5; Table 5). Sequence similarity threshold had little effect on final performance (Supplementary File S7, Table 5).

**TABLE 5:** Performance metrics of models trained on Baxter data and tested on Zeller data using a 97, 99 and 100% sequence similarity threshold. 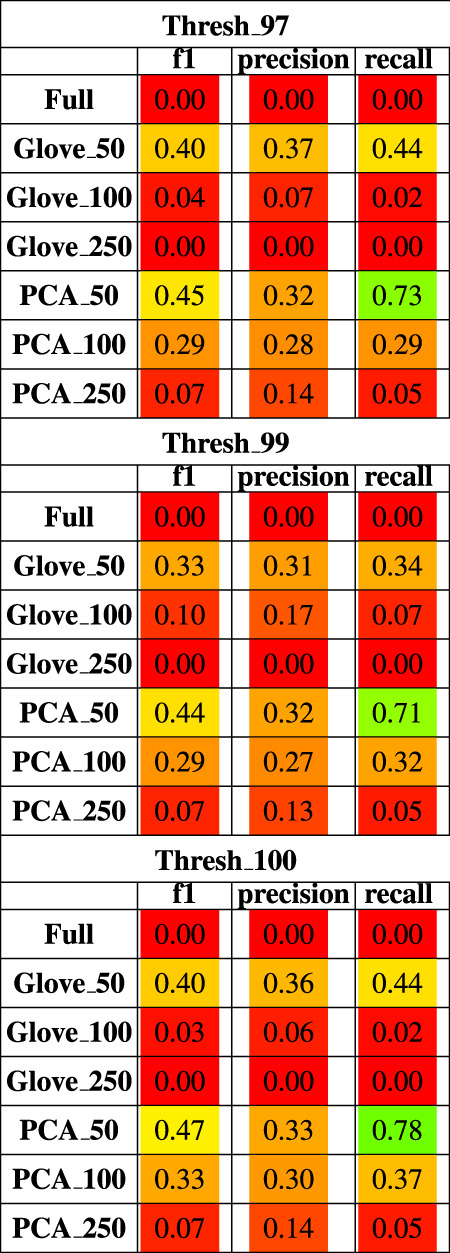

### 3.4 Metabolic Pathway Correlation

We correlated each embedding dimension with metabolic pathway genetic potential obtained from KEGG and PiCrust (See Methods). From this, we saw that dimensions all correlate to some groupings of metabolic pathways but not others (Supplementary Files S8–S13). This serves as a starting point in interpreting the biological functions of the otherwise mathematically defined dimensions in embedding space.

## 4 Discussion

16S studies often result in spurious associations between specific ASVs and host phenotype due to necessarily small sample sizes in comparison to feature spaces and the treatment of ASVs as independent features Schloss (2018), Ioannidis (2005), Fan et al. (2012). Embedding methods can address these issues by defining a new feature space, which can be thought of as combinations of ASVs, where ASVs are considered similar if they share co-occurrence or co-abundance patterns across a large dataset Pennington et al. (2014). Applying embedding methods to smaller datasets can increase the generalization of predictive classifiers that use gut microbiome data, and may lead to new insights about overarching microbial properties that independent ASV counts do not otherwise reflect Tataru and David (2020).

The embedding methods presented here are aimed to address the curse of dimensionality caused by a large number of variables (ASVs) measured across a relatively small number of samples. Machine learning models with too many input variables can easily overfit the training data, as observed with the normalized count data in this study. In addition, having too many input variables can saturate distance metrics, giving datapoints unique feature subsets that cause them to all appear equidistant Bai (2014). By reducing the dimensionality of the input data, we show that models are able to learn generalizable microbial patterns of disease and avoid overfitting on biomarkers specific to single datasets.

In the datasets tested, 50 dimensions offered the best, most consistently high performance on test set predictions. PCA-based transformation obtained higher recall without significant drop in precision as compared to GloVe-based transformation, but, in these datasets, both obtained considerably improved performance over the method of using normalized ASV counts. In most datasets, increasing the sequence similarity threshold did not affect generalizable performance significantly, with the exception of the HMP2 dataset where increasing threshold decreased recall significantly. This may be due to the relatively low number of original sequences utilized in embedding under the more stringent threshold.

### 4.1 Comparison to Other Work

Kubinski et al. tested machine learning predictive models using a leave one study out cross-validation across 15 IBD datasets that performed 16s sequencing on stool samples. Their random forest models obtained average F1 scores of 0.72 across studies when using species level annotations Kubinski et al. (2021). A study from Manandhar et al. also obtained a similar F1 score of 0.74 on a hold-on test portion of the American Gut Projet dataset Manandhar et al. (2021). These performances are just below the IBD testing results from this study using the Embed 50 and PCA50 models (F1 = 0.82–0.95) on HMP2 and Halfvarson datasets respectively. Interestingly, a study from Hassouneh et al. that combined metagenomic features (as opposed to 16s) and included 3 independent datasets obtained an F1 score 0.87, suggesting that perhaps the integration of multiple datasets into the training data combined with the use of non-amplicon microbiome features may lead to increased accuracy Hassouneh et al. (2021).

Wu et al. tested the predictive power of 16s microbiome features in predictive autism by annotating OTUs from five studies at the genus level, then applying a random forest model. When training on one dataset and testing on another, the models’ performance ranged from an F1 of 0.17–0.73 Wu et al. (2020). In comparison, the best performing model in the present study, GloVe_50, obtained an F1 of 0.68 on the testing data. Though they did not report F1 scores, other studies have reported surprisingly high values for area under the receiver operating curve when predicting autism (AUC = 0.93 and 0.98)Ding et al. (2020), Dan et al. (2020). This exceedingly high performance may be attributable to the sampling strategy, where ASD participants were recruited from the local hospital and typically developing participants from local kindergardens.

Wu et al. created a classifier that used fecal microbiome 16s sequences as well as age, sex, and BMI to distinguish patients with adenomas from colorectal cancer patients, and obtained an F1 score of 0.72. Models with equivalent hyperparameters and feature inputs trained on additional datasets also obtained F1 scores of 0.77 and 0.72) Wu et al. (2021). This is in line with the training F1 score obtained from the full model in this study from the Baxter data (F1 = 0.86) but higher than the training scores obtained from embedding methods (F1 = 0.58–0.68). Zhou et al. trained a random forest classifier to differentiate CRC from healthy controls using the same Baxter dataset presented in this study, and obtained an F1 score of 0.41, which is in the range of the F1 scores obtained here when testing the PCA50 model on an independent dataset (F1 = 0.43) Zhou et al. (2021). Neither of these studies tested their pre-trained models on independent datasets, so their true generalization capacity remains untested.

### 4.2 Limitations

This study used only the American Gut Project data to form the embedding transformation matrices. Integration of other, independent datasets would likely make the transformation process even more generalizable, especially to populations outside the United States.

In addition, Dada2 processing of reads and error model learning was performed on all the sequencing runs from the American Gut Project simultaneously in order to obtain one set of ASVs for all samples. This resulted in over 800,000 ASVs, most of which were not present in more than 10 samples. Learning an error model per sequencing run may have resulted in a lower rate of chimeric ASVs, which may have seen higher presence across samples Callahan et al. (2016).

While data transformed with either PCA or GloVe did provide grounds for more generalizable models, the interpretation of the learned representation remains a challenge. We find that correlations between learned taxa vector representations and metabolic pathway potential exist, however, each dimension correlates to a mixture of pathways, making direct implications difficult to conclude. In previous work, we found that mixtures of phylogenetic signal are also captured by learned dimensions Tataru and David (2020). Utilizing other natural language processing methods for dimensionality reduction like deep learning networks may allow us to take advantage of other interpretation methods like attention, saliency maps, or explanation generation to obtain a more complete understanding of the system Sun et al. (2021).

Lastly, the embedding matrices provided are specific to human gut microbiomes as measured from stool–embedding matrices for other biomes will be provided in future iterations.

## Data Availability

Publicly available datasets were analyzed in this study. This data can be found here: Halfvarson dataset: accession number PRJEB18471: https://www.ncbi.nlm.nih.gov/bioproject/PRJEB18471, HMP2 dataset: accession number PRJNA389280: https://www.ncbi.nlm.nih.gov/bioproject/PRJNA389280, Zeller dataset: accession number PRJEB18471: https://www.ncbi.nlm.nih.gov/bioproject/PRJEB18471, Baxter dataset: accession number PRJNA290926: https://www.ncbi.nlm.nih.gov/bioproject/PRJNA290926, M3 dataset: https://files.cgrb.oregonstate.edu/David_Lab/M3/, Pilot dataset: https://files.cgrb.oregonstate.edu/David_Lab/M3_longitudinal_16s.
