# Supplementary figures and images for: GMEmbeddings: An R Package to Apply Embedding Techniques to Microbiome Data
Source: Front Bioinform. 2022 Apr 26;2:828703. doi: 10.3389/fbinf.2022.828703 (PMC9580954; doi:10.3389/fbinf.2022.828703)

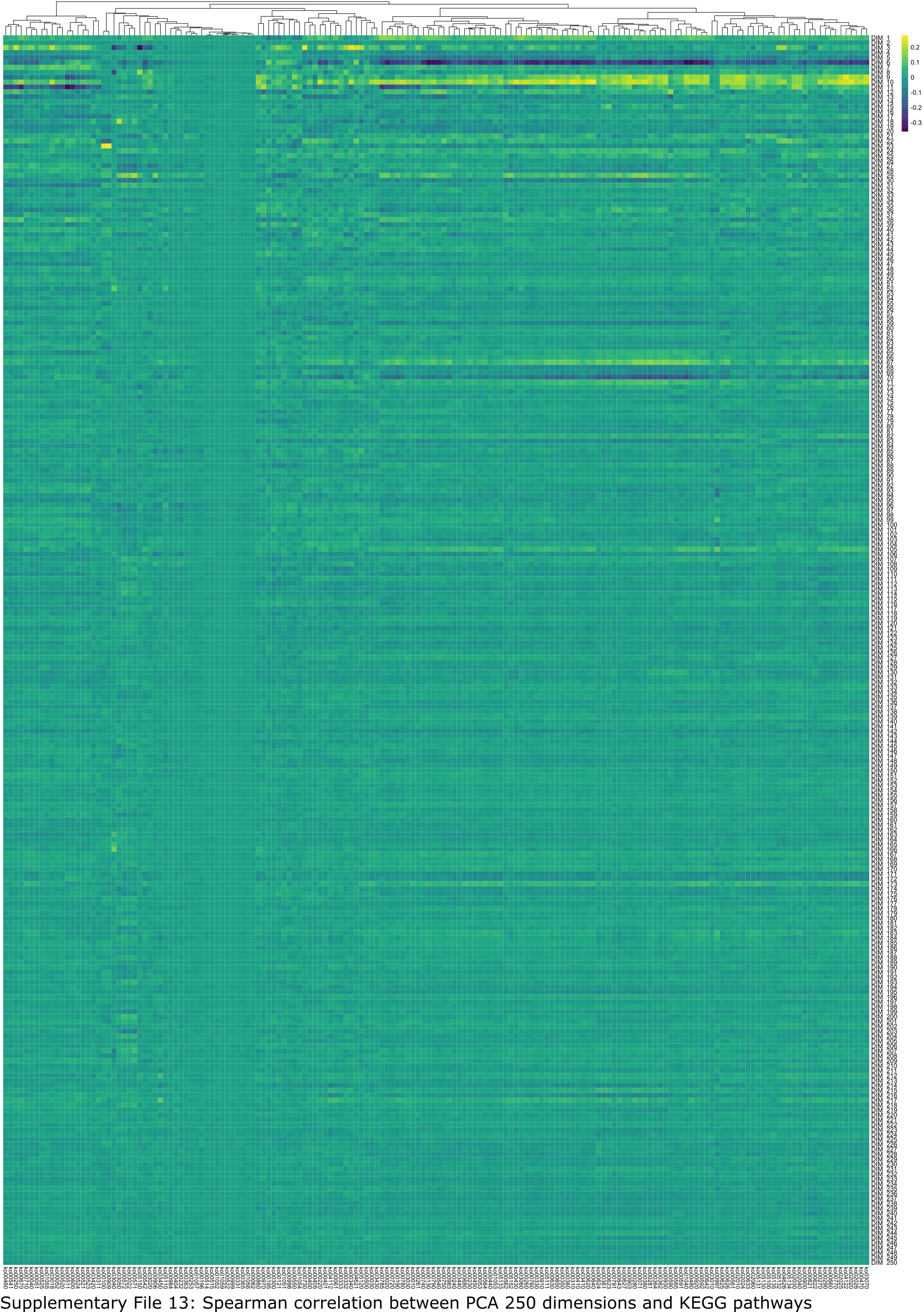

Supplement: Supplementary file 1 [file DataSheet1.ZIP › Supplementary13_pca_250dim_corr_pathways.jpg]

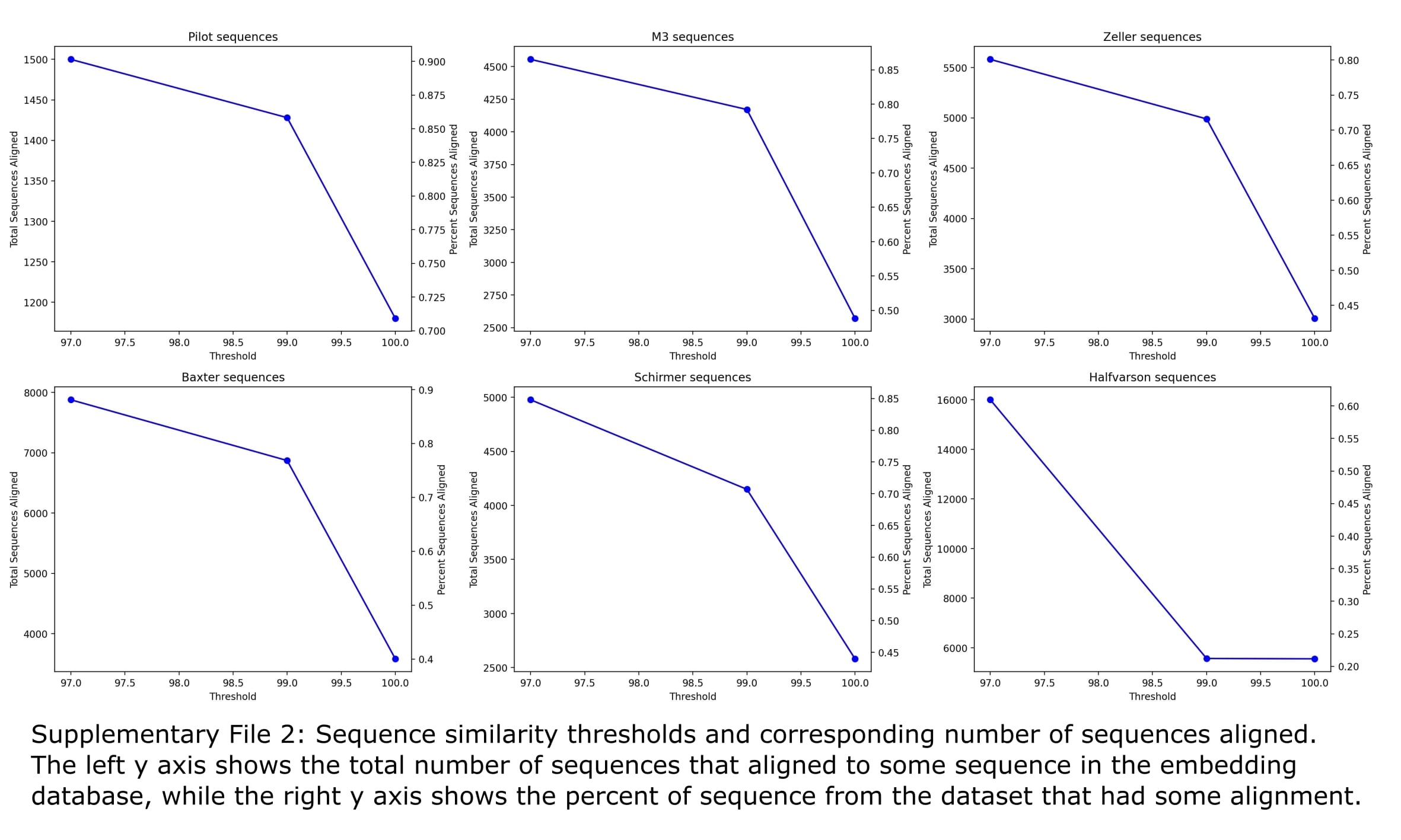

Supplement: Supplementary file 1 [file DataSheet1.ZIP › Supplementary2_query_sequences_aligned.jpg]

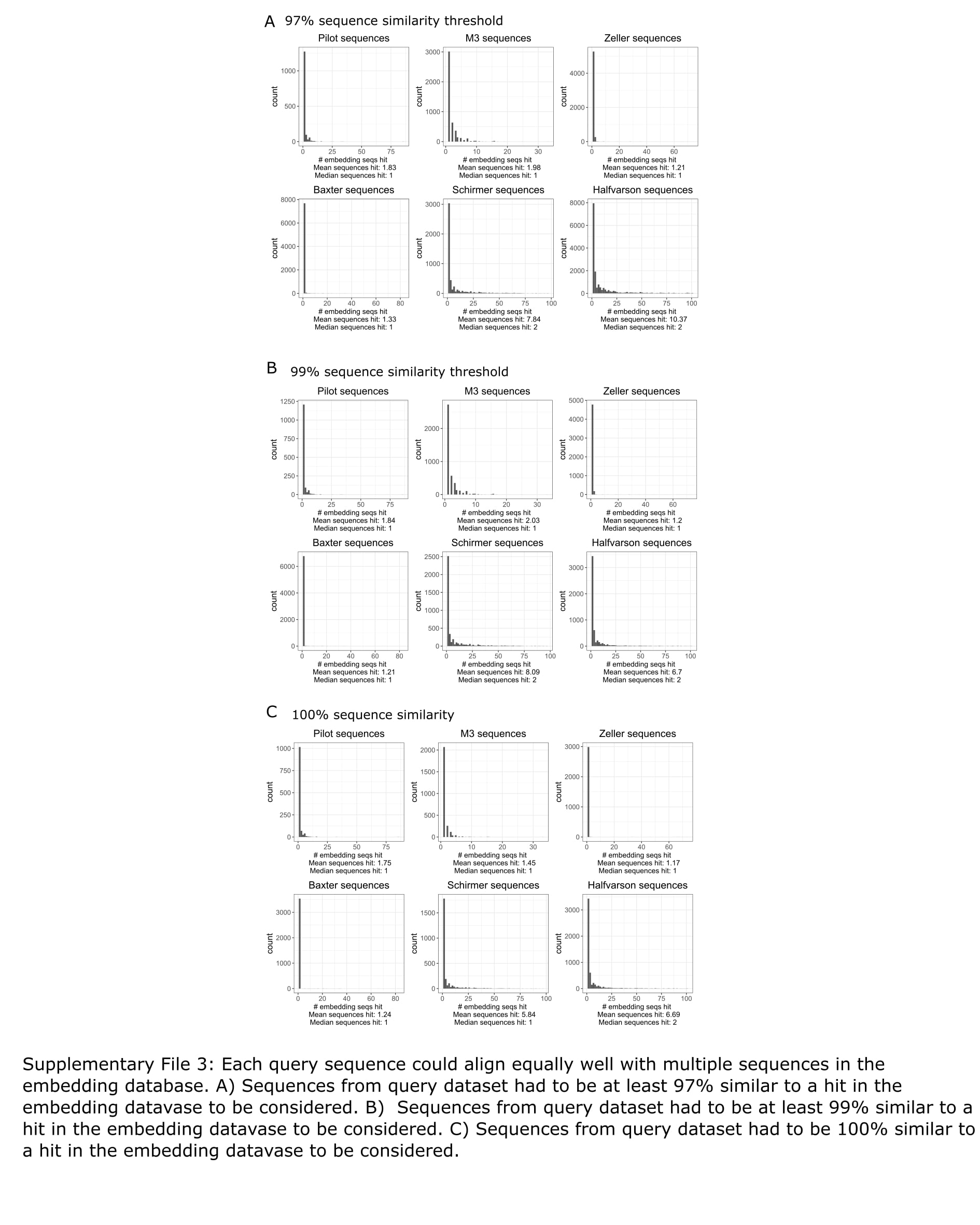

Supplement: Supplementary file 1 [file DataSheet1.ZIP › Supplementary3_embedding_sequences_hit.jpg]

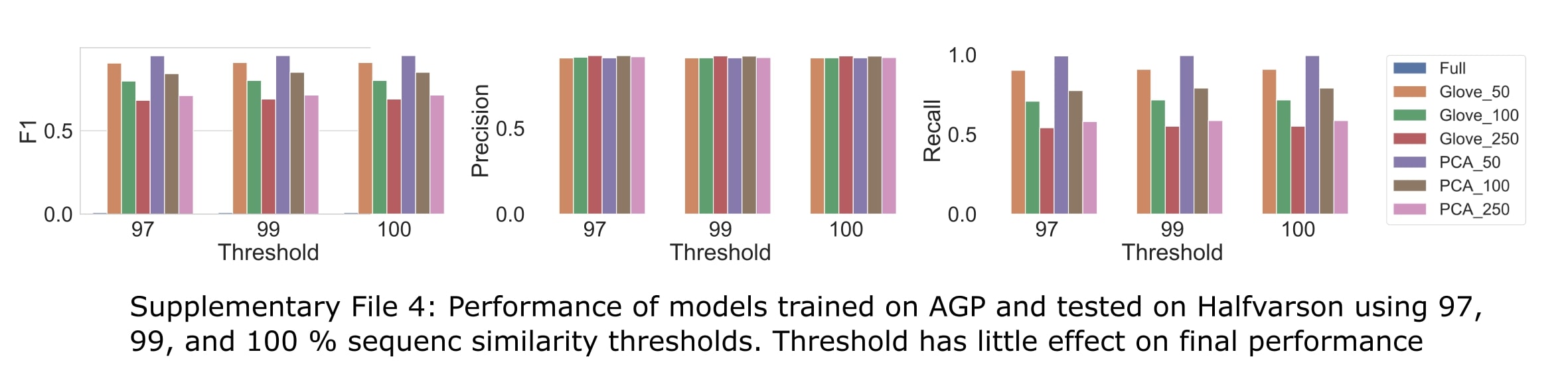

Supplement: Supplementary file 1 [file DataSheet1.ZIP › Supplementary4_perf_halfvarson.jpg]

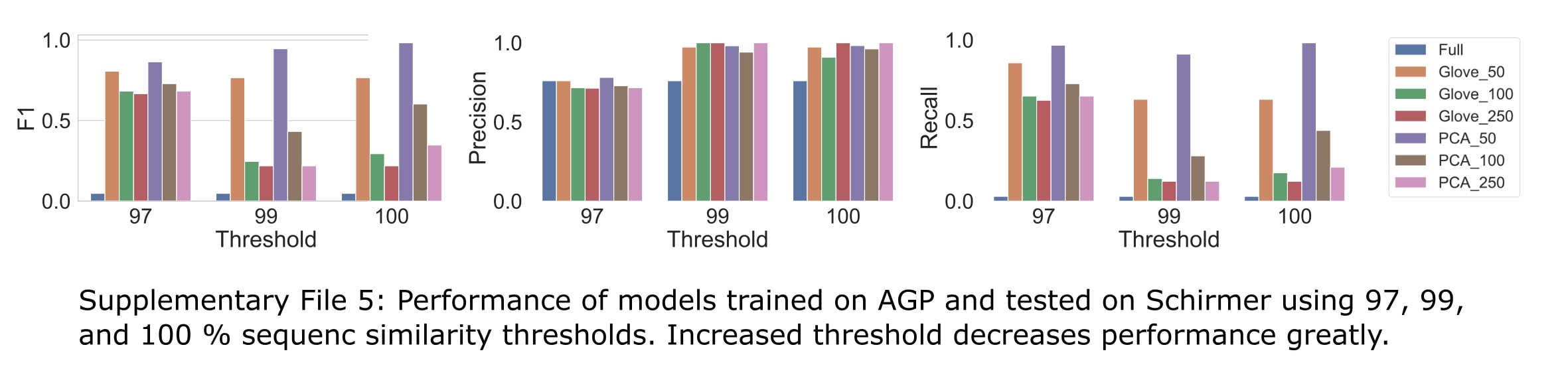

Supplement: Supplementary file 1 [file DataSheet1.ZIP › Supplementary5_perf_schirmer.jpg]

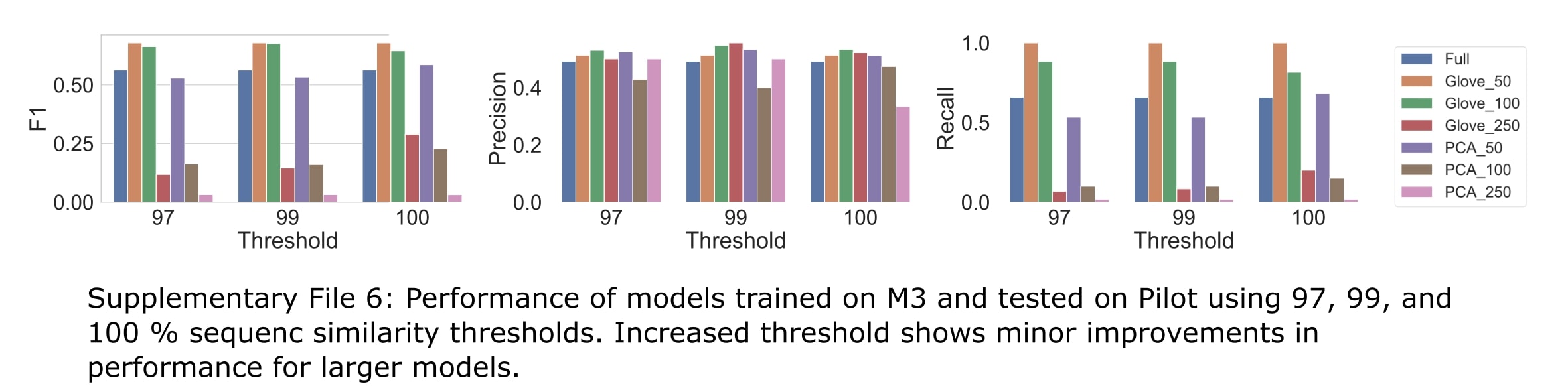

Supplement: Supplementary file 1 [file DataSheet1.ZIP › Supplementary6_perf_autism.jpg]

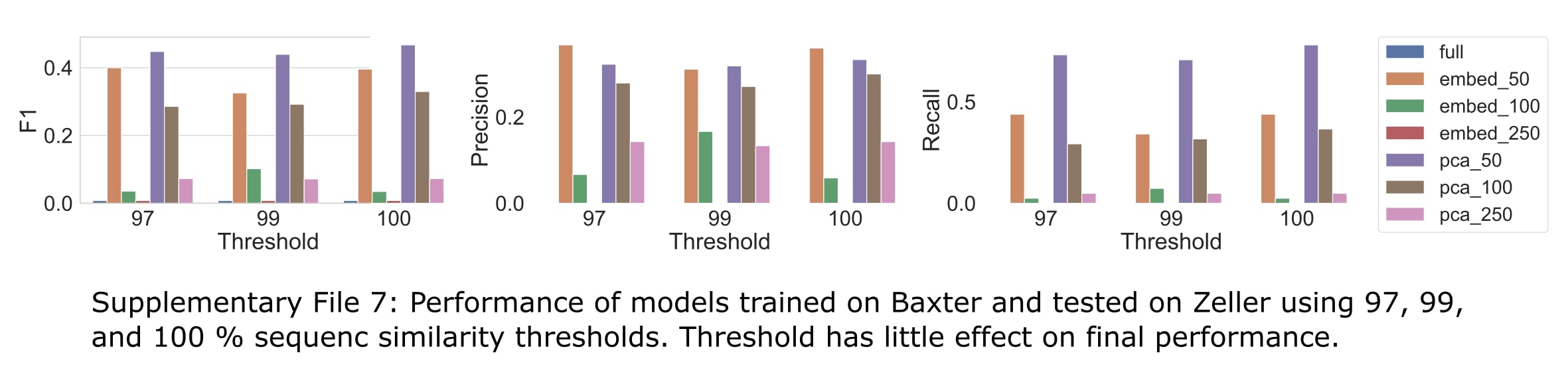

Supplement: Supplementary file 1 [file DataSheet1.ZIP › Supplementary7_perf_crc.jpg]

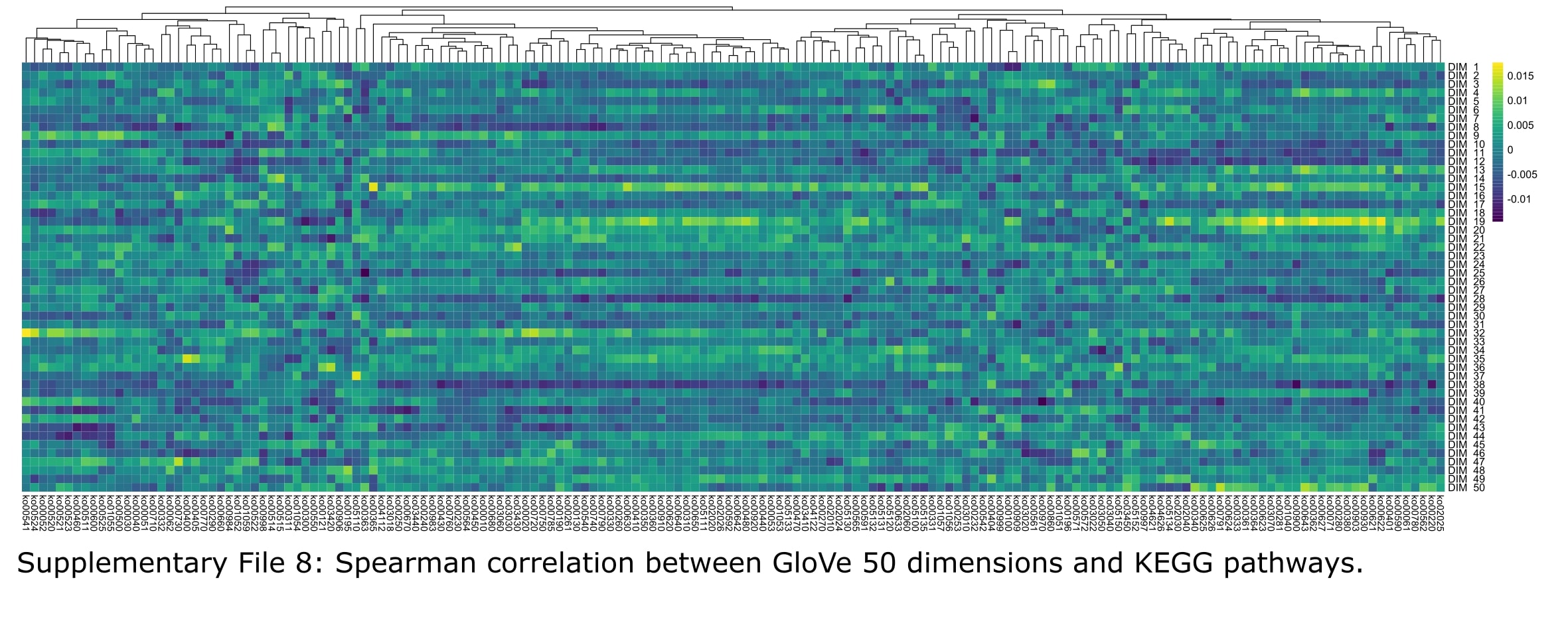

Supplement: Supplementary file 1 [file DataSheet1.ZIP › Supplementary8_glove_50dim_corr_pathways.jpg]

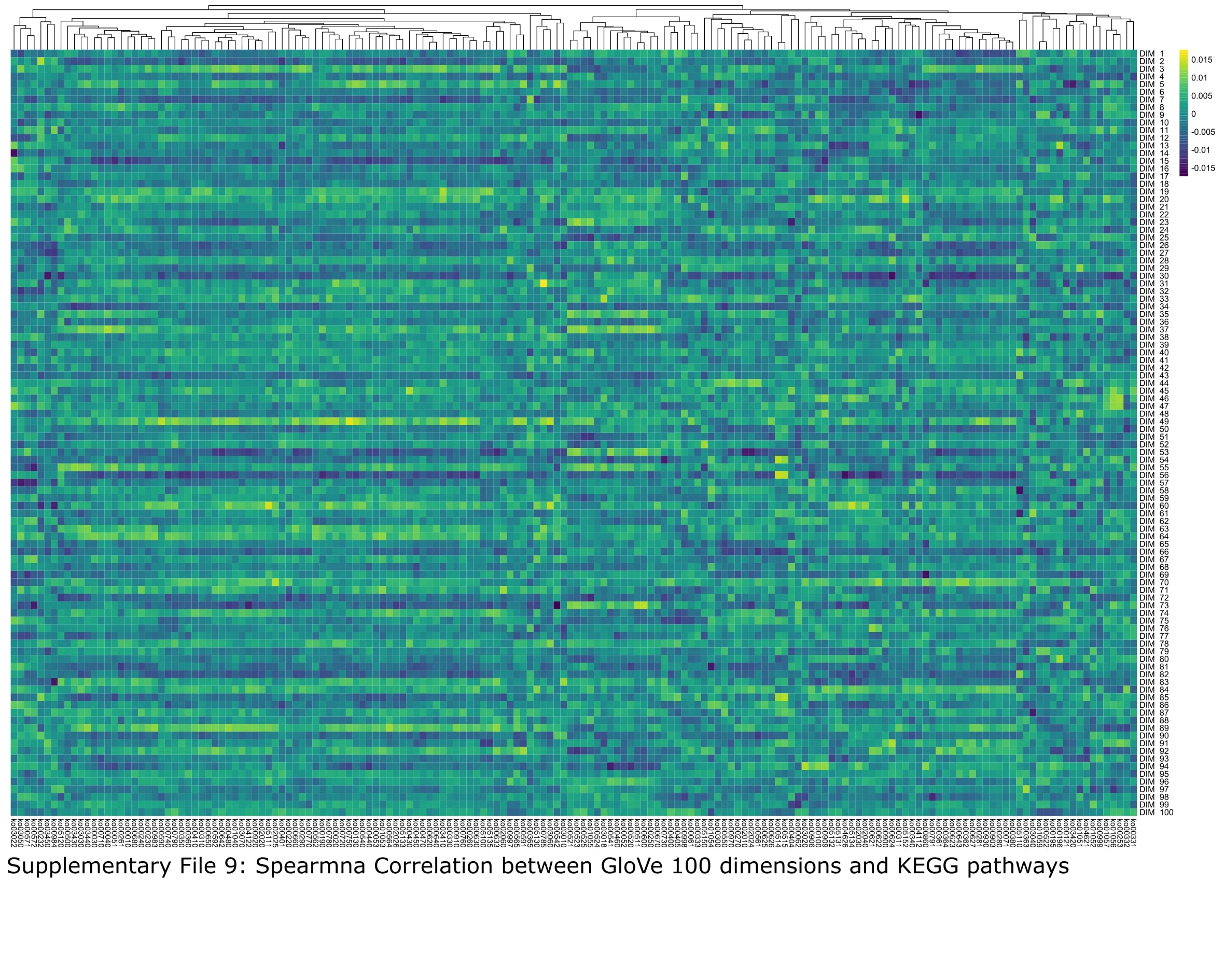

Supplement: Supplementary file 1 [file DataSheet1.ZIP › Supplementary9_glove_100dim_corr_pathways.jpg]

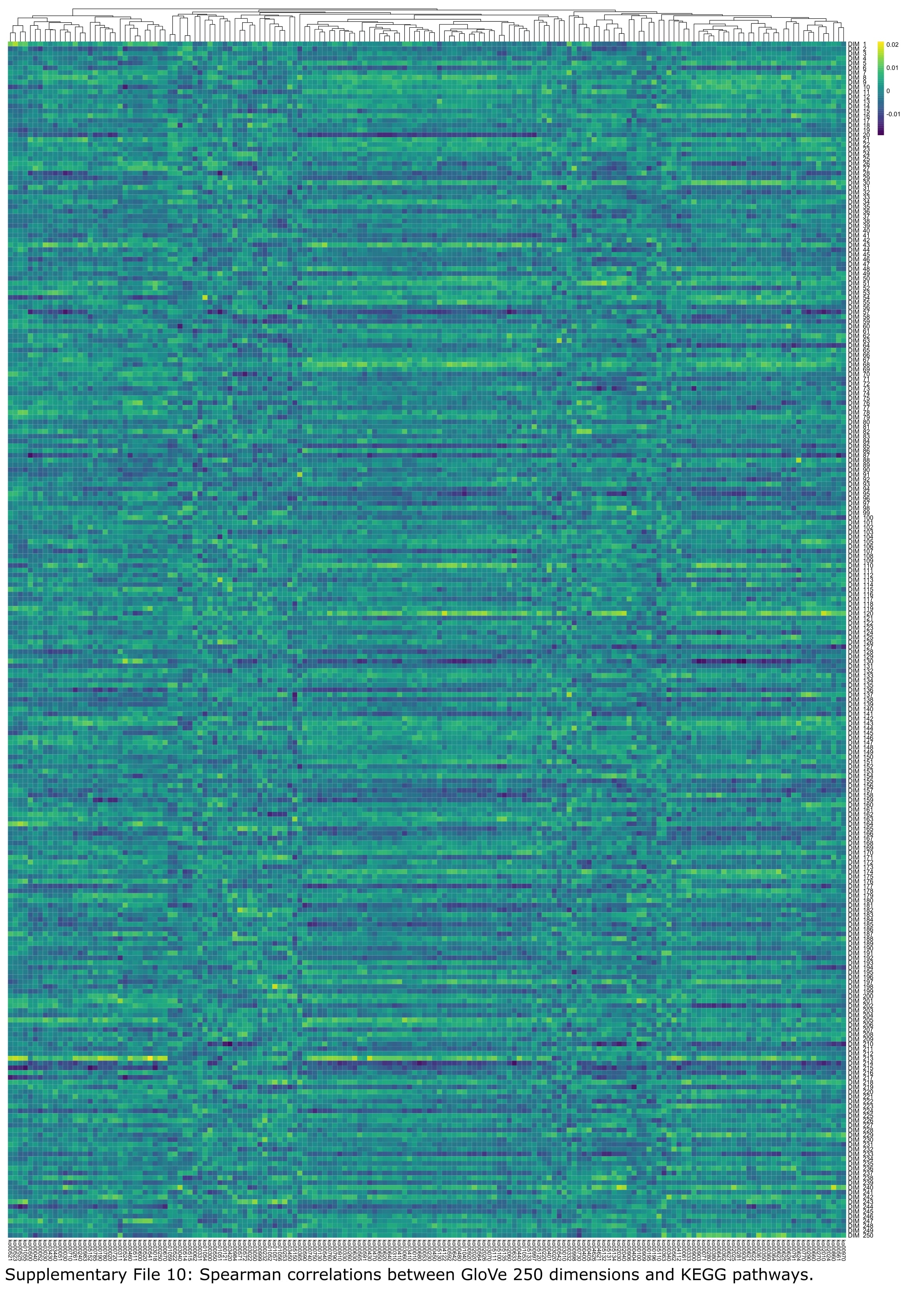

Supplement: Supplementary file 1 [file DataSheet1.ZIP › Supplementary10_glove_250dim_corr_pathways.jpg]

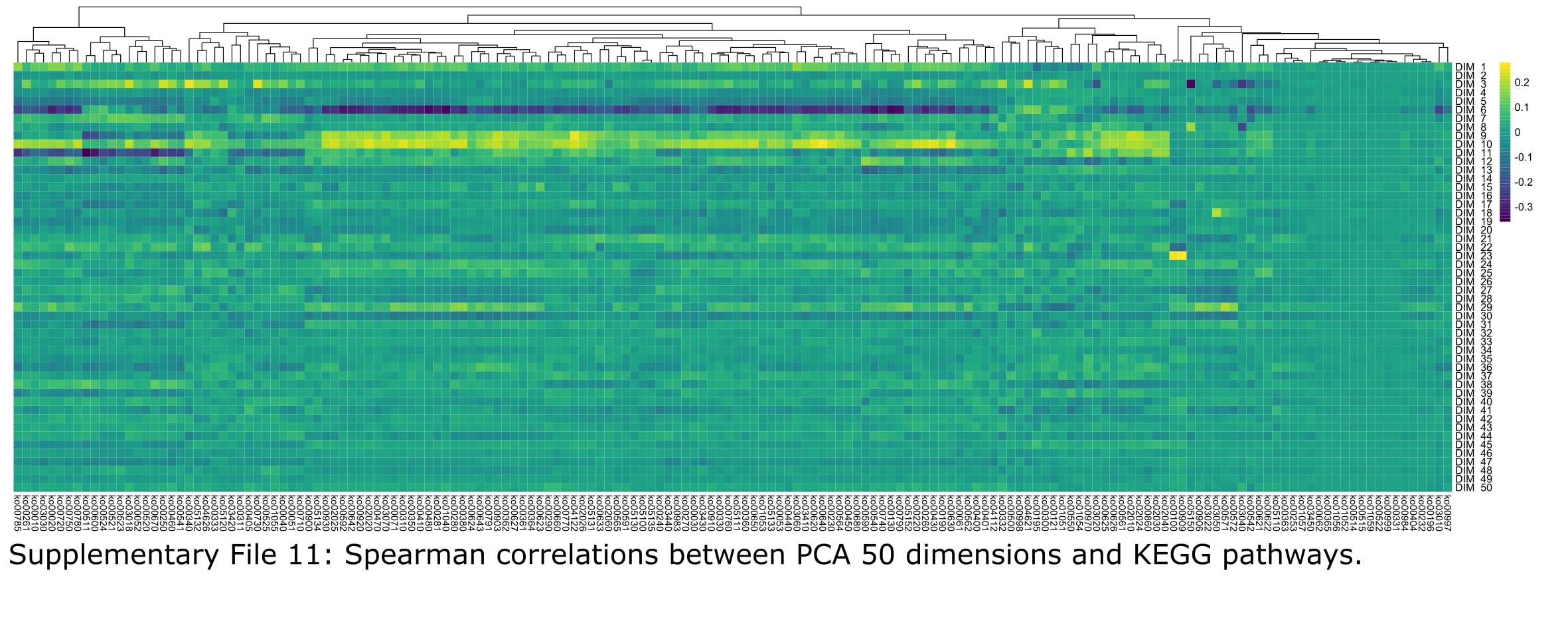

Supplement: Supplementary file 1 [file DataSheet1.ZIP › Supplementary11_pca_50dim_corr_pathways.jpg]

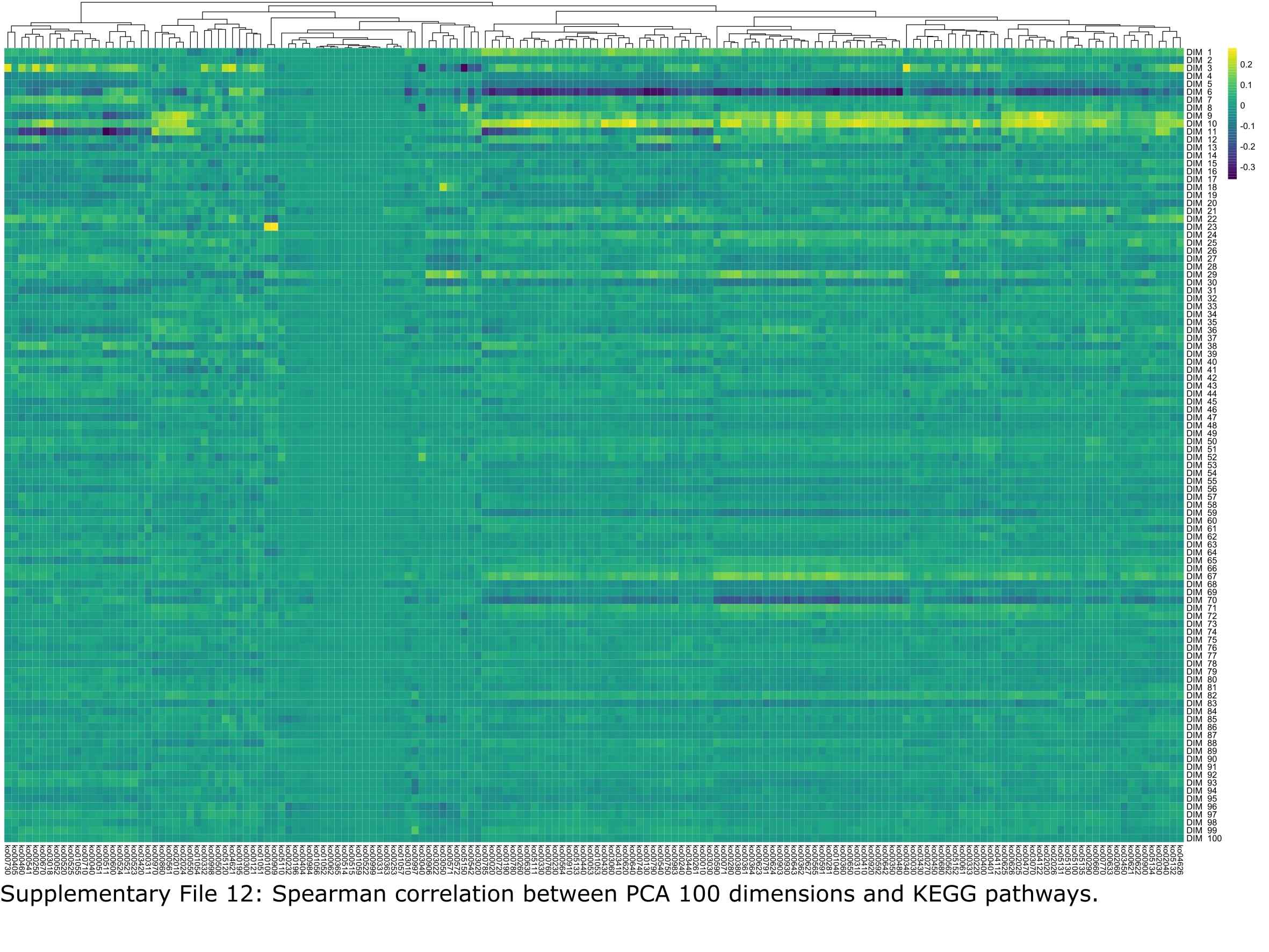

Supplement: Supplementary file 1 [file DataSheet1.ZIP › Supplementary12_pca_100dim_corr_pathways.jpg]

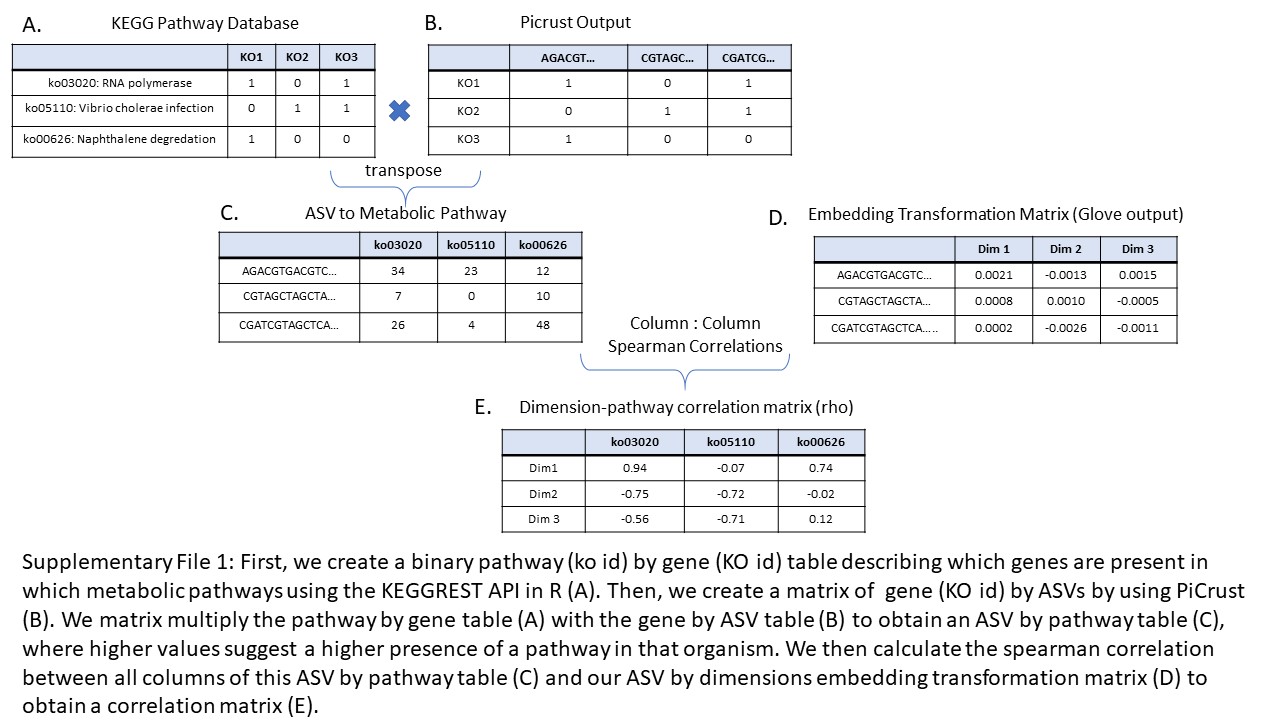

Supplement: Supplementary file 1 [file DataSheet1.ZIP › Supplementary1_metabolic_correlation_process_infographic.jpg]
